# Arthropod prey vary among orders in their nutrient and exoskeleton content

**DOI:** 10.1002/ece3.8280

**Published:** 2021-12-14

**Authors:** Jamie T. Reeves, Samuel D. Fuhlendorf, Craig A. Davis, Shawn M. Wilder

**Affiliations:** ^1^ Department of Integrative Biology Oklahoma State University Stillwater Oklahoma USA; ^2^ Department of Natural Resource Ecology and Management Oklahoma State University Stillwater Oklahoma USA

**Keywords:** indigestible components, insectivore, lipid, prey quality, protein

## Abstract

Insectivores gain macronutrients and elements from consuming arthropod prey, but must also deal with indigestible components (i.e., exoskeleton) of prey. For example, avian chicks (e.g., northern bobwhites; *Colinus virginianus*) have limited gut space, and ingesting prey with relatively higher proportions of indigestible components may impact assimilation efficiency, growth, and survival. The ability of insectivores to choose higher quality prey would depend on prey taxa varying consistently in nutritional content. We tested whether there were consistent differences among taxonomic orders of arthropod prey in their macronutrient (protein and lipid), elemental (C and N), and exoskeleton content. We used northern bobwhite chicks as our focal insectivore and focused on their potential prey as a case study. We also tested the influence of indigestible exoskeleton on the measurement of macronutrient content and the ability of elemental content to predict macronutrients. We found large and consistent variation in macronutrient and elemental content between arthropod orders. Some orders had consistently high protein content and low exoskeleton content (i.e., Araneae) and are likely higher quality prey for insectivores. Abundant orders common in the diets of insectivores, like Hymenoptera and Coleoptera, had high exoskeleton content and low protein content. We also found support for the ability of elements to predict macronutrients and found that metabolizable (i.e., exoskeleton removed) elemental content better predicted macronutrient content. A better understanding of arthropod nutrient content is critical for elucidating the role of spatial and temporal variation in prey communities in shaping the growth and survival of insectivores.

## Graphical abstract

We tested whether there were consistent differences in macronutrient, elemental, and exoskeleton content among taxonomic orders of potential arthropod prey of northern bobwhite chicks. We found large and consistent variation between orders, with orders consistently low in exoskeleton content exhibiting high metabolizable protein content (i.e., Araneae). We also found that orders consistently high in exoskeleton exhibited low metabolizable protein content.

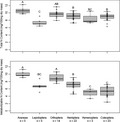

## 1 INTRODUCTION

Arthropods are an essential food source for a wide variety of invertebrates and vertebrates (Butler et al., 2012; Durst et al., 2008; Kaspari & Joern, 1993a,1993b; Uetz et al., 1992). Generalist insectivores often consume a diversity of prey that can vary widely in quality. Arthropod prey provide bulk nutrients such as carbohydrates, lipids, and protein that are important as a source of energy and for building body mass (Eubanks & Dimmick, 1974; Giuliano et al., 1996; Harveson et al., 2004; Nestler et al., 1942). However, the considerable variation in nutrient content observed among arthropod taxa (10%–85% protein and 5%–32% lipid by dry mass) can result in changes to nutrient assimilation by consumers depending on diet breadth and the relative abundance of prey (Wilder et al., 2013). Past studies have identified particular prey species that are high or low quality due to their nutritional or defensive compound content (Lease & Wolf, 2010; Lease & Wolf, 2011; Razeng & Watson, 2014; Wilder, 2011). Yet, less is known about consistency or variation within and among arthropod orders in their nutritional content. Consistency of nutritional content within orders of arthropods could form an evolutionary basis through which predators could base prey choice decisions and may allow better understanding of how spatial and temporal variation in prey communities affect the distribution of nutrients across the landscape and their availability to opportunistic predators.

In addition to macronutrients, exoskeleton may also be an important dietary consideration for insectivorous taxa. Exoskeleton is often a significant component of arthropod bodies and can vary among taxa, with exoskeleton comprising 18%–60% of dry mass (Lease & Wolf, 2010). Arthropod exoskeleton consists largely of chitin (20%–50%), but can have considerable amounts of protein locked within the chitinous matrix during sclerotization (Lease & Wolf, 2010). Hence, exoskeleton can contain significant amounts of both carbon and nitrogen. Yet, chitin digestibility is quite variable across consumers. Chitinase production appears conserved among many mammals, though much of the available evidence has only identified whether or not taxa produce chitinase and where production occurs and has not considered the effect of chitinase production on the degree or efficiency of digesting chitin in the diets of these species (Cornelius et al., 1975; Jeuniaux, 1961; Strobel et al., 2013; Whitaker et al., 2004). For example, large quantities of exoskeleton fragments are common in bat dung and can be used to identify prey, suggesting that bats do not efficiently digest or assimilate chitin (Whitaker, 1995). Chitin digestibility is also particularly variable across avian taxa, as seabirds are capable of digesting biologically meaningful amounts of chitin (39.1%–84.8% of ingested chitin across seabird taxa; Jackson et al., 1992), while generalist raptors digest less on average (screech owls 10.6%–30.4%; American kestrel 15.7%–25.7%; Akaki & Duke, 1999). American robins (7.8%–13.6% digestibility) and Northern bobwhites (6.7% digestibility) also have low digestion of chitin (Weiser et al., 1997). In addition, most predatory arthropods do not even ingest chitin when feeding on prey as they use extraoral digestion (Cohen, 1995). Hence, the protein, carbon, and nitrogen in exoskeleton are likely unavailable to many insectivores because they cannot digest significant amounts of chitin (Akaki & Duke, 1999; Bell, 1990; Weiser et al., 1997).

In addition to affecting assimilation by consumers, exoskeleton can affect the measurement of prey nutrient content. For example, a common measure of arthropod nutrient content (i.e., crude protein = 6.25 × total nitrogen) assumes that all nitrogen is available to consumers and does not account for variation in the quantity and digestibility of exoskeleton in prey (Jones, 1941; Peoples, 1992; Peoples et al., 1994; Razeng & Watson, 2014). Use of different measures of nutrients can lead to different conclusions. For example, measures of the crude protein content of beetles have suggested that they have high protein content (Razeng & Watson, 2014), while colorimetric assays of protein have suggested that beetles have low metabolizable protein content (Wilder et al., 2013). Because generalist insectivores feed opportunistically and changes in prey abundance influence consumption, it is important to consider how the relative proportions of digestible and indigestible arthropod tissues influence prey quality, nutrient availability for predators, and the way that nutrients in prey are measured (Lease & Wolf, 2010; Wilder et al., 2013, 2019).

A variety of vertebrate and invertebrate insectivores rely on arthropods for most or all of their diet. For example, northern bobwhites (*Colinus virginianus*; hereafter bobwhite) are seasonally insectivorous, and the proportion of arthropods in the bobwhite diet depends on sex and life stage (Butler et al., 2012; Doxon & Carroll, 2010; Eubanks & Dimmick, 1974; Foye et al., 2015). Brooding hens require large amounts of arthropod‐derived protein and energy in order to produce high‐quality eggs (Giuliano et al., 1996; Harveson et al., 2004), and chicks require a high‐protein (~28%), arthropod‐based diet (94.1% up to two weeks post hatch) to quickly accumulate mass and develop feathers necessary for locomotion and predator avoidance (Eubanks & Dimmick, 1974; Foye et al., 2015; Giuliano et al., 1996; Harveson et al., 2004; Nestler et al., 1942; Scott et al., 1963). In the present study, we use bobwhites as a focal insectivore because the digestibility of exoskeleton is known and low for this species (Weiser et al., 1997) and because their nutrient requirements, prey size preference, and capture limitations are well‐documented (Butler et al., 2012; Doxon & Carroll, 2010; Eubanks & Dimmick, 1974; Foye et al., 2015). Hence, this allows us to focus our analysis on a group of prey that are all relevant to a consumer as opposed to using the entire community of prey some of which may or may not be consumed by a single consumer species. While we focus our analysis on bobwhites, the results of this study are likely relevant to a wide range of insectivores with similar prey size preferences.

We collected ground‐dwelling arthropod prey of bobwhites to (1) test if different taxonomic orders of arthropods vary consistently in nutrient content in terms of macronutrients (lipid and protein), exoskeleton, and elements (C and N) and (2) test the strength of correlations between elements (i.e., C and N) and macronutrients (lipids and protein) in potential prey. Additionally, we partitioned the elements into those in the exoskeleton (i.e., indigestible) and those in the rest of the body (i.e., metabolizable) to test if total or metabolizable elemental content was more closely related to macronutrient content of prey.

## 2 METHODS

### 2.1 Study site

The arthropods used in this study were collected at Packsaddle Wildlife Management Area in Ellis County, Oklahoma, during the months of May, June, and July 2019. Annual rainfall is 63.5 cm on average. Packsaddle WMA contains a wide variety of soil types including fine sandy loams, loam fine sands, and fine sands (Oklahoma Dept. of Wildlife Conservation and the United States Department of Agriculture). The 6475‐ha WMA is managed with prescribed fire, strip disking, and cattle grazing primarily for the production of game birds such as bobwhites and common turkey (*Meleagris gallopavo*), but many other vertebrate and invertebrate insectivores inhabit the area for a significant portion of the year. Common vegetation present at sites includes grasses such as big bluestem (*Andropogon gerardii*), Indian grass (*Sorghastrum nutans*), little bluestem (*Schizachyrium scoparium*), side‐oats grama (*Bouteloua curtipendula*), and buffalo grass (*Bouteloua dactyloides*), as well as shrubs like shinnery‐oak (*Quercus havardii*), sand sagebrush (*Artemisia filifolia*), and sandplum (*Prunus angustifolia*; Oklahoma Department of Wildlife Conservation).

### 2.2 Invertebrate collection and identification

The goal of invertebrate collection for this study was to collect as diverse of a sample of potential prey of bobwhite quail as possible. Invertebrates common in the bobwhite diet based on crop analyses include members of the orders *Hymenoptera*, *Coleoptera*, *Hemiptera*, *Orthoptera*, *Araneae*, and *Lepidoptera* (Butler et al., 2012; Doxon & Carroll, 2010; Eubanks & Dimmick, 1974). Invertebrates were collected in three, 5‐day sampling periods in May, June, and July 2019 using sweep net, dry pitfall, coverboard, and hand collection techniques (Doxon et al., 2011; Taylor & Brereton, 1995). Sweep net samples were collected in burned, strip‐disked, and unmanaged areas using 40‐m transects, and a total of 20 sweep net samples were collected per sampling period. Collection locations were not evenly distributed across the landscape but were located in areas of diverse topography and vegetative cover. Four 1‐m square coverboards were deployed in one burned, one disked, and two unmanaged areas. Transects of five dry pitfall traps were placed in one burned, one disked, and two unmanaged areas. Coverboard and dry pitfall trap samples were collected twice daily (morning and evening), and 1 h was spent searching for and hand collecting invertebrates daily. All samples were stored in plastic bags and frozen until sorting.

Individual invertebrates were sorted out of plant matter and other debris and were initially sorted based on taxonomic order. Individuals were then given a morphospecies label based on differences in appearance, and representatives of each morphospecies were pinned in a reference collection. Individuals of each morphospecies were pooled across collection dates and locations. The number of morphospecies per order used in this study was related to sample availability and an attempt to avoid over‐ or underrepresentation of taxa relative to their known biodiversity. In total, we measured the nutrient content of the following morphospecies: 23 Coleoptera, 22 Hemiptera, 3 Hymenoptera (all ants; bees and wasps were excluded), 14 Orthoptera, 5 larval Lepidoptera, and 5 Araneae.

### 2.3 Nutrient analyses

Two identical sets of 72 samples (i.e., same morphospecies) were prepared for exoskeleton and nutrient analysis, respectively, by drying samples for 24 h at 60°C and measuring their dry mass. We sorted 15–30 mg of dry mass for each sample, with the number of individuals per sample varying based on the body size of the arthropods and availability of biomass for each morphospecies across the sampling season. For example, some Orthoptera samples were only 1–2 individuals, while ant samples contained as many as 30 individuals, and some Hemiptera and Coleoptera contained biomass from individuals collected across all three sampling months. Macronutrient and exoskeleton content was measured according to established methods that were combined into a standardized protocol (Cuff et al., 2021), which are summarized here. We measured lipid content of arthropods using a gravimetric method with chloroform as a solvent. All dried samples were soaked in chloroform for 48 h (Wilder et al., 2013). Chloroform was removed and new chloroform was added every 24 h, and samples were then dried for 24 h at 60°C and reweighed (Lease & Wolf, 2011; Wilder et al., 2013). Exoskeleton was removed from one set of samples by soaking in 0.1 M NaOH to dissolve soft tissue (Lease & Wolf, 2010). Samples were first sonicated at 80°C in 0.1 M NaOH for 30 min and then allowed to soak for 24 h. After 24 h, samples were centrifuged at 11,200 *g*, the NaOH was removed, and fresh NaOH was added. After another 24 h, samples were centrifuged again and the NaOH was removed, and samples were washed with water and dried at 60°C for 24 h. The dry weight after soft tissue removal was used as a measure of exoskeleton (Lease & Wolf, 2010).

Samples of 2–3 mg of ground, lipid‐free arthropod tissue, as well as one sample of exoskeleton for each order of arthropods, were also prepared for elemental C and N content analysis. Samples were weighed on a microbalance and packaged in tin capsules to be combusted in an Elementar. Metabolizable elemental content was considered to be the elements in the part of the body that was not exoskeleton.

Protein content of samples was also measured using colorimetric assays on each morphospecies in which there was sufficient biomass remaining. Protein was extracted from arthropods by grinding lipid‐free samples with a 3‐mm steel ball‐bearing using a mixer mill at 30 hz for 3 min. Then, approximately 5 mg of ground arthropod material was soaked in 1 ml of 0.1 M NaOH and sonicated at 80°C for 30 min. The supernatant was then used to conduct the Lowry assay and the Bradford assay according to the kit instructions for microplate assays. Bovine IgG standard solutions were used to create standard curves.

### 2.4 Data analysis

Statistical program R ver 3.4.2 (R Core Team, 2013) was used to conduct one‐way ANOVAs and Tukey's HSD post hoc analysis to detect differences in lipid, exoskeleton, protein, and elemental content between orders of arthropods. Levene's test was used to test for homogeneity of variance. When the assumption of homogeneity of variance was not met, we performed Welch's ANOVA and the Games–Howell post hoc test. Linear regression was used to test the relationship between elemental content and macronutrient content, and Akaike's information criterion for small sample sizes (AICc) was used to compare the predictive ability of total and metabolizable measures of elemental content. Nutrient content of arthropods is expressed as mg/100 mg dry mass to use units that are independent of body size.

## 3 RESULTS

### 3.1 Among‐ and within‐order variation in content

#### 3.1.1 Exoskeleton content

**FIGURE 1:**
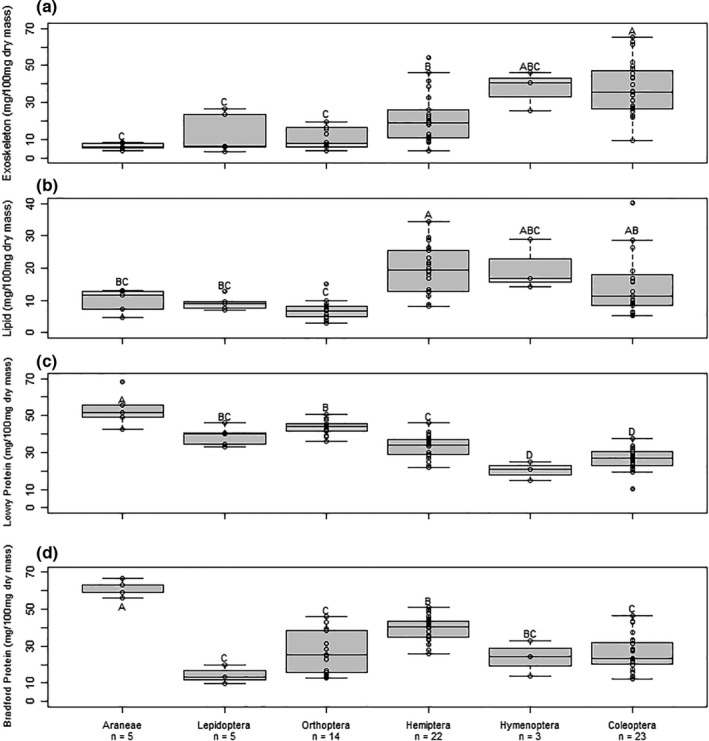
(a) Exoskeleton, (b) lipid, (c) Lowry protein, and (d) Bradford protein content of six arthropod orders as a proportion of total dry mass (mg/100 mg dry mass). Orders not connected by the same letter are significantly different (*p* < .05). Boxes represent upper and lower quartiles, midlines represent medians, and whiskers represent nonoutlier maxima and minima. Any points outside of whiskers are outliers

Mean exoskeleton content differed among orders (Table 1a; Figure 1a). There were also differences in variance among treatments with Coleoptera exoskeleton content being more variable than any other order (Levene's test, *p* = .01). Welch's ANOVA, which we conducted due to unequal variances among groups, indicated that exoskeleton content differed significantly between orders of arthropods (*p* < .001) (Table 1a; Figure 1a). Araneae had the lowest mean exoskeleton content, and Coleoptera and Hymenoptera had the highest, although Hymenoptera did not differ significantly from any order likely due to the small sample size of this group (Table 1a; Figure 1a). The mean exoskeleton contents of Hymenoptera and Coleoptera were ~6 times higher than Araneae. Orthoptera and Lepidoptera did not differ significantly from Araneae, and Hemiptera had intermediate exoskeleton content (Table 1a; Figure 1a).

**TABLE 1:** Mean and standard deviation values for macronutrient and elemental content of arthropods of six orders

Order	(a) Exoskeleton		(b) Lipid		(c) Lowry Protein		(d) Bradford Protein	
	Mean	SD	Mean	SD	Mean	SD	Mean	SD
Araneae	6.2	0.8	10.0	1.7	53.4	4.2	60.9	1.9
Coleoptera	37.5	3.0	14.5	1.9	26.5	1.2	26.4	2.0
Hemiptera	21.7	2.9	19.5	1.5	33.2	1.3	39.4	1.4
Hymenoptera	37.4	6.1	20.1	4.5	20.3	2.9	23.8	5.5
Lepidoptera	13.1	5.0	9.3	1.0	38.7	2.3	14.4	3.0
Orthoptera	10.6	1.6	7.1	0.8	43.4	1.1	26.6	3.1

	(e) Total C		(f) Metabolizable C		(g) Total N		(h) Metabolizable N	
Araneae	43.9	1.1	41.2	0.9	10.7	0.4	10.0	0.4
Coleoptera	48.5	0.4	31.2	1.5	8.9	0.2	5.4	0.3
Hemiptera	48.1	0.4	38.1	1.5	9.0	0.2	7.1	0.3
Hymenoptera	47.7	0.07	33.5	2.3	8.5	1.0	4.8	0.8
Lepidoptera	40.9	0.7	36.3	2.0	7.5	0.5	6.8	0.6
Orthoptera	44.6	0.5	40.1	0.9	9.6	0.3	8.9	0.3

Note

All data are presented as mg/100 mg dry mass.

#### 3.1.2 Lipid content

Mean lipid content also differed among orders (Table 1b; Figure 1b). There were differences in variance among treatments with Hemiptera lipid content being more variable than any other order (Levene's test; *p* = .008). Welch's ANOVA indicated that lipid content differed significantly between orders of arthropods (*p* < .001). Hymenoptera and Hemiptera had the highest average lipid content (Table 1b; Figure 1b). The average lipid content of Hymenoptera and Hemiptera was at least double Araneae, Lepidoptera, and Orthoptera lipid content (Table 1a; Figure 1a). Coleopterans were intermediate, with significantly higher lipid content than Orthoptera (*p* < .05; Table 1b; Figure 1b). Araneae, Lepidoptera, and Orthoptera had the lowest average lipid content (Table 1b; Figure 1b).

#### 3.1.3 Protein content

The Lowry assay suggested that mean protein content differed between orders of arthropods (*p* < .0001; Table 1c; Figure 1c). Levene's test failed to detect differences among taxa in variance of protein content measured by the Lowry assay (*p* = .5). Araneae had the highest protein content, and Coleoptera and Hymenoptera had the lowest (Table 1c; Figure 1c). Orthoptera, Lepidoptera, and Hemiptera had intermediate protein content, though Lepidoptera protein content did not differ from Orthoptera or Hemiptera (Table 1c; Figure 1c).

The Bradford assay also suggested that there were large differences in protein content between orders of arthropods (*p* < .0001; Table 1d; Figure 1d). However, where the Lowry assay produced distinct differences between intermediate and low‐protein orders, the Bradford assay placed Orthoptera lower in rank and grouped Orthoptera, Lepidoptera, Coleoptera, and Hymenoptera as the lowest in protein content. Levene's test failed to detect differences among taxa in variance of protein content measured by the Bradford assay (*p* = .07). Araneae had the highest protein content, and Orthoptera, Lepidoptera, Coleoptera, and Hymenoptera had the lowest (Table 1d; Figure 1d). Hemiptera had intermediate protein content, though it was not significantly different from Hymenoptera (Table 1d; Figure 1d).

#### 3.1.4 Total elemental content

**FIGURE 2:**
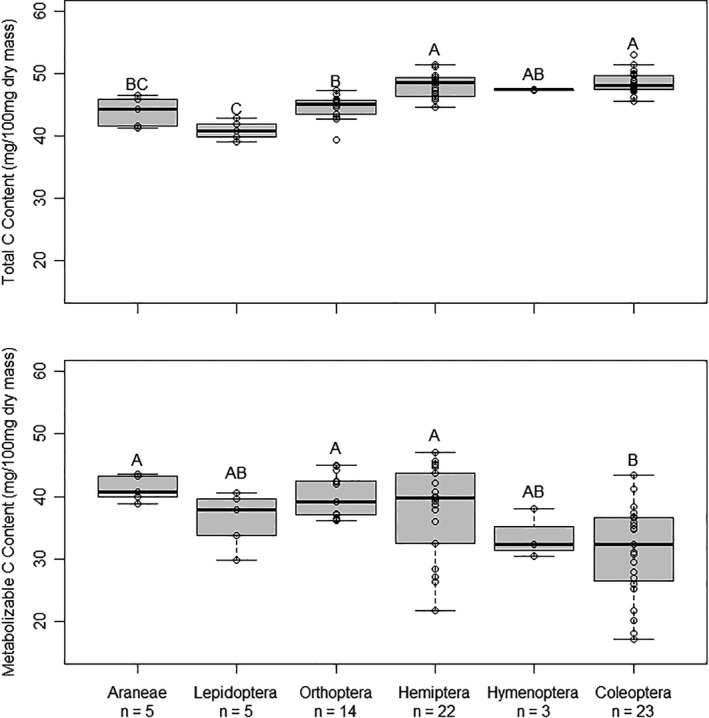
Total and metabolizable C content of six arthropod orders as a proportion of dry mass. Orders not connected by the same letter are significantly different (*p* < .05). Boxes represent upper and lower quartiles, midlines represent medians, and whiskers represent nonoutlier maxima and minima. Any points outside of whiskers are outliers

C and N content also differed between orders of arthropods, though mean C content was somewhat less variable between orders than mean N content. Levene's test failed to detect differences in variance of total C between orders (*p* = .3; Figure 2). However, total C content differed significantly between orders (*p* < .0001; Table 1e; Figure 2). Lepidoptera had the lowest average total C content, and Hemiptera, Coleoptera, and Hymenoptera had the highest (Table 1e; Figure 2). Orthoptera had intermediate total C content, and Araneae total C content was not significantly different from any other order (Table 1e; Figure 2).

**FIGURE 3:**
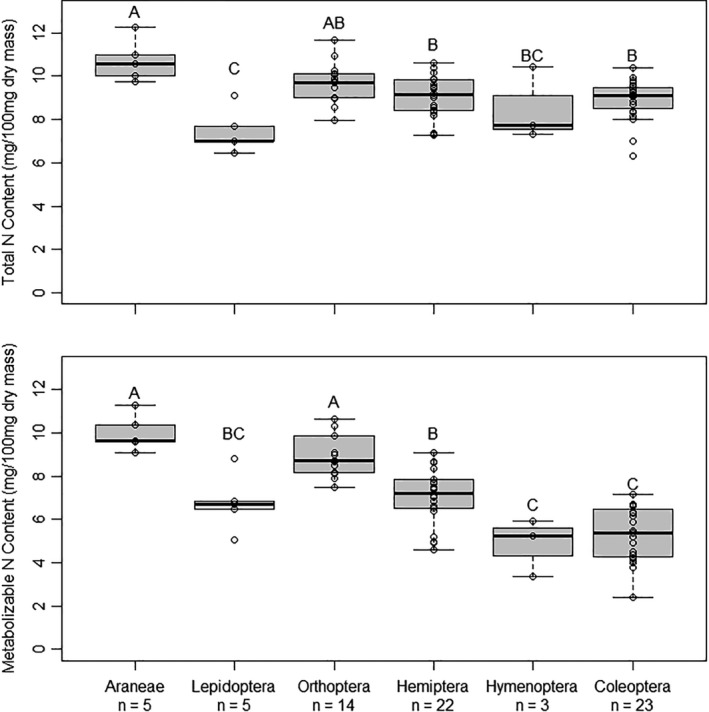
Total and metabolizable N content of six arthropod orders as a proportion of dry mass. Orders not connected by the same letter are significantly different (*p* < .05). Boxes represent upper and lower quartiles, midlines represent medians, and whiskers represent nonoutlier maxima and minima. Any points outside of whiskers are outliers

Levene's test failed to detect differences among taxa in variance of total N (*p* = .7). There were significant differences among Orders in total N (*p* = .01). Lepidoptera had the lowest average total N content and Araneae had the highest (Table 1g; Figure 3). There was a gradient in total N among taxa, with taxa ranked highest to lowest as Araneae, Orthoptera, Hemiptera, Coleoptera, Hymenoptera, and Lepidoptera (Table 1g; Figure 3).

#### 3.1.5 Metabolizable elemental content

Patterns in metabolizable C content were different than total C, particularly for orders with high exoskeleton content (i.e., Coleoptera; Table 1f; Figure 2). Levene's test indicated that variance in metabolizable C differed between orders (*p* = .02), and Coleoptera adults had the most variable metabolizable C content (Figure 2). Welch's ANOVA indicated that metabolizable C differed between orders (*p* = .003; Table 1f; Figure 2). Araneae, Orthoptera, Hemiptera, and Lepidoptera had the highest average metabolizable C content, and Coleoptera adults had the lowest (Table 1f; Figure 2). Hymenoptera did not significantly differ from any other order (Table 1f; Figure 2).

When we analyzed metabolizable N (i.e., total N with exoskeleton N removed), the rank of some orders changed relative to the results for total N, though we still observed significant different between orders (*p* < .0001; Table 1h; Figure 3). Levene's test failed to detect differences among taxa in variance of metabolizable N (*p* = .8). Araneae had the highest average metabolizable N content (Table 1h; Figure 3). The mean metabolizable N content of Araneae was approximately double that of the lowest two orders: Coleoptera adults and Hymenoptera (Figure 3). Orthoptera had similar metabolizable N content to Araneae, but was only significantly higher than Coleoptera and Hymenoptera (Figure 3). Hemiptera was intermediate, but was only significantly lower than Araneae and higher than Coleoptera (Table 1h; Figure 3). Lepidoptera, Coleoptera, and Hymenoptera had the lowest metabolizable N content (Figure 3).

### 3.2 Elemental and macronutrient relationships

#### 3.2.1 Relationships between C and C‐containing compounds

**FIGURE 4:**
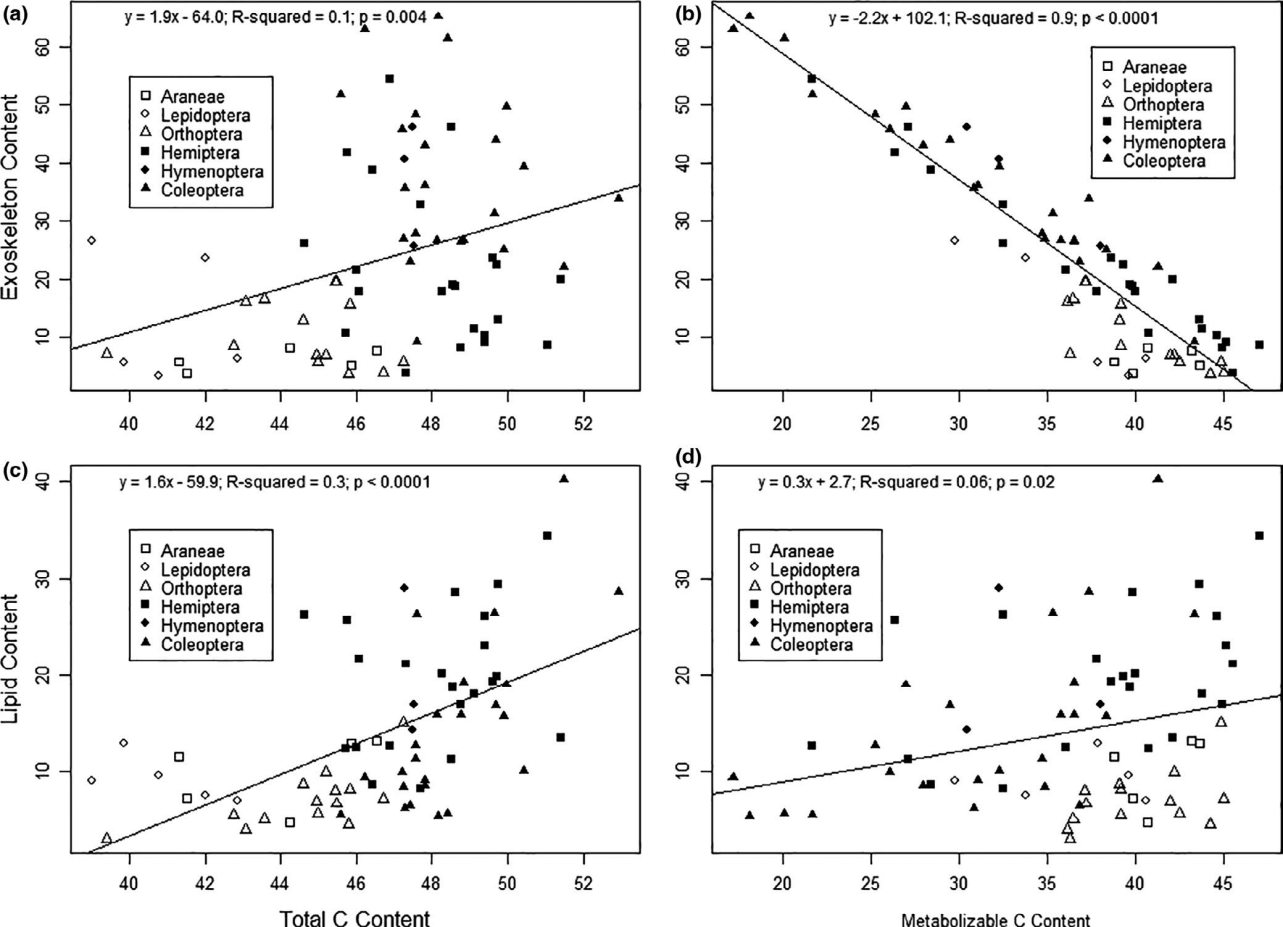
Linear models of exoskeleton content predicted by (a) total C and (b) metabolizable C, and lipid content predicted by (c) total C and (d) metabolizable C content of six arthropod orders. All values are displayed in mg/100 mg dry mass

Total C content was positively related to exoskeleton content (*R* ^2^ = .1; *p* = .004; Figure 4a). However, total C poorly accounted for variation in the exoskeletal data (Figure 4a). Metabolizable C displayed a negative linear relationship with exoskeletal content (*R* ^2^ = .9; *p* < .0001; Figure 4b). AICc model selection indicated that metabolizable C was a much better predictor of exoskeleton content than total C (Table 2). Metabolizable C was the top model (delta AICc = 0.00) and total C received considerably less support in its predictive ability of exoskeleton content (delta AICc = 136.24; Table 2). No individual Order displayed a significant relationship between total C and exoskeleton (Table S1a), but all Orders except Araneae displayed significant, negative relationships between metabolizable C and exoskeleton (Table S1b).

**TABLE 2:** AICc model selection of linear models of exoskeleton, lipid, and protein content (Lowry and Bradford)

Model Response	Predictor	K	AICc	delta AICc	Model Weight	Cumulative Weight
Exoskeleton	Metabolizable C	3	468.05	0.00	1	1
	Total C	3	604.29	136.24	0	1
	Null	2	610.55	142.50	0	1
Lipid	Total C	3	484.56	0.00	1	1
	Metabolizable C	3	506.76	22.20	0	1
	Null	2	510.10	25.54	0	1
Lowry Protein	Metabolizable N	3	494.67	0.00	1	1
	Total N	3	530.01	35.35	0	1
	Null	2	541.76	47.10	0	1
Bradford Protein	Metabolizable N	3	547.98	0.00	0.93	0.93
	Total N	3	553.19	5.21	0.07	1
	Null	2	566.97	18.99	0	1

Note

Exoskeleton and lipid content were predicted using total and metabolizable C, and protein content was predicted using total and metabolizable N. Models within delta AICc < 2.00 are considered to receive equal support. Delta AICc values were calculated relative to the model with the lowest AICc value.

Total C content was also positively related to lipid content (*R* ^2^ = .3; *p* < .0001; Figure 4c). However, the low *R* ^2^ value suggests there is much unexplained variation (Figure 4c). Metabolizable C also displayed a positive linear relationship with lipid content (*R* ^2^ = .06; *p* = .02; Figure 4d). Lipid content was better predicted by total C content than metabolizable C (Table 2). The model with total C as the predictor was the top model (delta AICc = 0.00) and received more support in its ability to predict lipid content than the metabolizable C model (delta AICc = 22.20; Table 2). Though lipid content linearly increased with total C content across all Orders, only Coleoptera displayed a significant relationship between lipid and total C content (Table S1c). However, Coleoptera and Hemiptera individually displayed significant, positive relationships between lipid content and metabolizable C (Table S1d).

#### 3.2.2 Relationships between N and Protein

**FIGURE 5:**
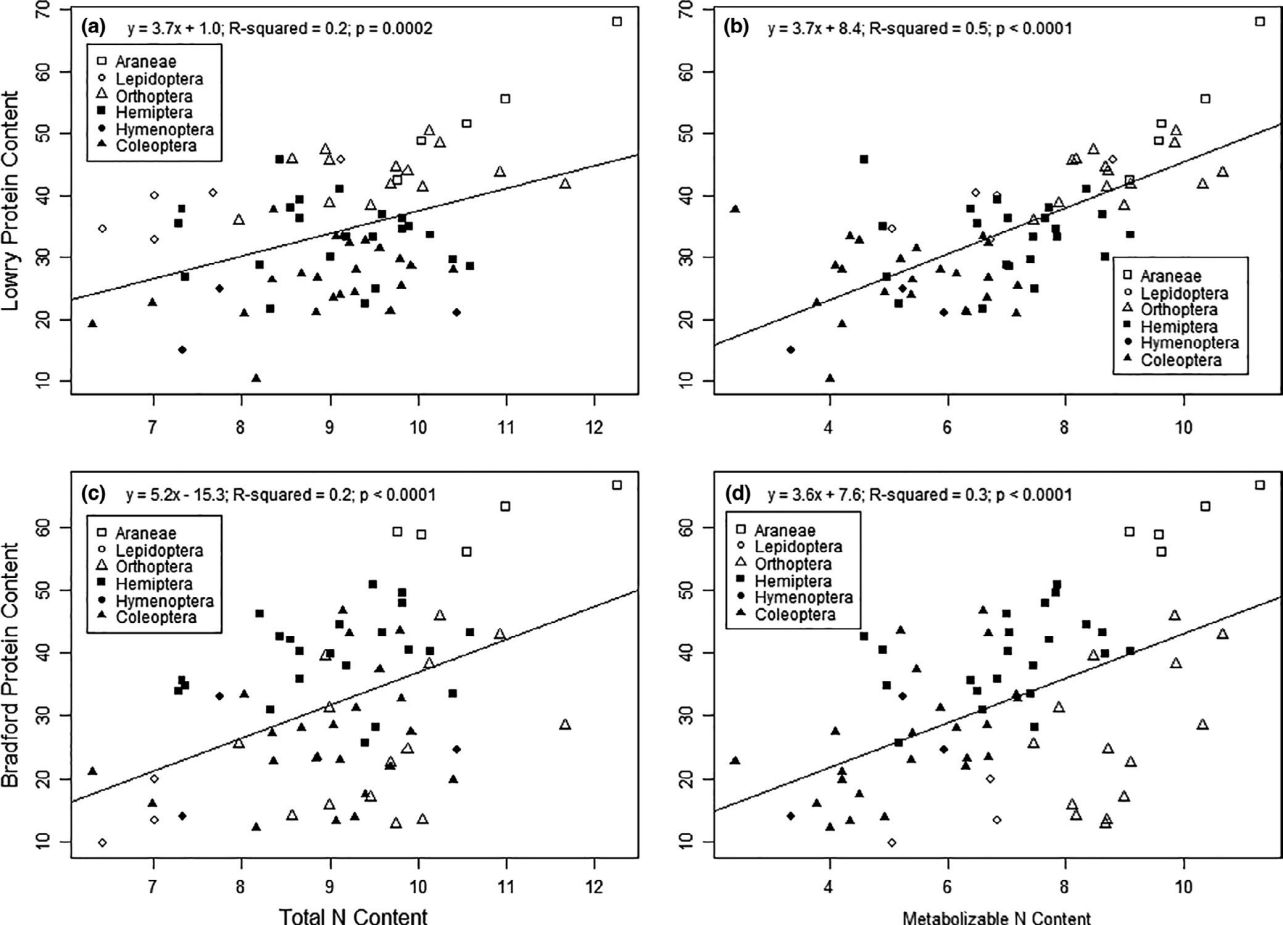
Linear models of Lowry protein content predicted by (a) total N and (b) metabolizable N, and Bradford protein content predicted by (c) total N and (d) metabolizable N content of six arthropod orders. All values are displayed in mg/100 mg dry mass

The Lowry assay displayed a weak correlation with total N (*R* ^2^ = .2; *p* = .0002; Figure 5a) and a stronger correlation between (*R* ^2^ = .5; *p* < .0001; Figure 5b). Metabolizable N content was a better predictor of the Lowry assay than total N (Table 2). The model containing metabolizable N as the predictor for Lowry protein content received considerably more support (delta AICc = 0.00) than the model using total N as the predictor (delta AICc = 35.35; Table 2). There were also significant, positive relationships between total N and Lowry protein content in Araneae, Lepidoptera, and Coleoptera (Table S1e), though only Araneae displayed a significant, positive relationship between metabolizable N and Lowry protein content (Table S1f).

The Bradford assay displayed a slightly higher correlation between metabolizable N (*R* ^2^ = 0.3; *p* < .0001) than total N (*R* ^2^ = .2; *p* < .0001; Figure 5c,d). AICc model selection indicated that metabolizable N predicted Bradford protein content better than total N (Table 2). The top model contained metabolizable N as the predictor (delta AICc = 0.00), and the total N model received less support (delta AICc = 5.21; Table 2). Additionally, no orders displayed relationships between total N and Bradford protein content (Table S1g), but Araneae, Orthoptera, and Coleoptera displayed significant, positive relationships between metabolizable N and Bradford protein content (Table S1h).

## 4 DISCUSSION

We observed substantial differences in elemental and macronutrient content between Orders of common arthropods. Overall, our results support the hypothesis that arthropod taxa are consistently different from each other in nutrient content, although some taxa are more variable in nutrient content than others. Araneae had the highest protein content and lowest exoskeleton content, although Orthoptera also had high protein content and Lepidoptera also had low exoskeleton content. In contrast, Coleoptera and Hymenoptera had the highest exoskeleton content and lowest protein content. Hymenoptera and Hemiptera had the highest lipid content, although they were also the most variable. Large, consistent differences in macronutrient content between Orders of arthropods may provide predators with the opportunity to select prey to regulate their nutrient intake and indicate that changes in the frequency of Orders in the diets of predators can affect their nutrient intake (Bell, 1990; Weiser et al., 1997; Wilder et al., 2019).

Variation in nutrient content within orders of arthropods may also have important consequences for consumers. Adult beetles are extremely variable in body form and nutritional composition (McCullough et al., 2015; Sloggett, 2008), and we found that Coleoptera exoskeleton content was the most variable of any order. Coleoptera and Hemiptera also displayed consistently large within‐order variation in exoskeleton and lipid content. Thus, it appears that some orders are highly variable in nutrient content, particularly in nutrients contributing to pools of C, where other orders (i.e., Araneae, Orthoptera, Lepidoptera) and macronutrient/elemental pools (i.e., protein/N) remained fairly consistent. For the taxa with higher within‐Order variation, it would be interesting to know whether lower levels of taxonomic identification (e.g., Family or Genus) produced groups that were less variable in nutrient composition.

There was a lot of variation in the strengths of the relationships between elements and macronutrients. Total C was a better predictor of lipid content, and metabolizable C was a better predictor of exoskeleton content, although the relationship was inverse (i.e., arthropods with higher metabolizable C had less exoskeleton). The relationship between nitrogen and protein was also supported. Metabolizable N predicted protein content measured by both the Bradford and Lowry assays better than total N. These results suggest that metabolizable N can be a predictor of protein content and potential nutrient intake of insectivores, likely because metabolizable N excludes indigestible exoskeletal content (Bell, 1990; Weiser et al., 1997; Wilder et al., 2019). Other preliminary data suggest that metabolizable N may be significantly correlated with metabolizable amino acid content of samples, which is considered to be one of the most accurate but most expensive measures of protein content (S.M. Wilder and C.L. Barnes, Unpublished results) and thus may provide an inexpensive proxy for amino acid content.

Many consumers cannot digest exoskeleton in meaningful quantities, and it is therefore essential to consider how indigestible components of prey influence potential nutrient intake (Akaki & Duke, 1999; Cohen, 1995; Weiser et al., 1997; Wilder et al., 2019). For example, two of the most common arthropod orders (Coleoptera and Hymenoptera; in terms of abundance at our study site; Reeves et al. (In revision), as well as abundance in the bobwhite diet in other studies; Eubanks & Dimmick, 1974; Butler et al., 2012) had the highest mean exoskeleton content of all orders (Butler et al., 2012; Doxon & Carroll, 2010). Individuals likely gain greater nutritional benefits when consuming prey low in exoskeleton due to increases in assimilation efficiency (Nestler et al., 1942; Peoples et al., 1994). Hence, changes in the relative abundance of high (e.g., Coleoptera and Hymenoptera) versus low (e.g., Araneae) exoskeleton prey could have important impacts on overall nutrient intake by insectivores (Butler et al., 2012; Foye et al., 2015; Morrow et al., 2015; Weiser et al., 1997). While some insectivores are able to modulate expenditures related to handling indigestible components (i.e., extraoral digestion in spiders avoids ingestion of exoskeleton; Cohen, 1995), many consumers do not have these adaptations and cannot digest exoskeleton. It is thus critical to consider how ingestion of indigestible components impact consumers, as food limitation can result in deficiencies in assimilation, growth, development, locomotor ability, reproduction, and ultimate survival (Gregg & Rogers, 1986; Kaur & Ab, 2015; Peoples, 1992; Peoples et al., 1994).

Another finding of our comparison of nitrogen and protein was that the method of estimating protein content of arthropods had significant impacts on the results. Our results suggested that the Lowry assay and metabolizable N provided similar patterns of results in estimated nutrient content of arthropods. Total N has commonly been used to calculate crude protein, which is N × 6.25 (Bukkens, 1997; Finke, 2013, 2015; Jones, 1941). Yet, our results suggested that there can be considerable differences between total N and metabolizable N for some taxa, especially Coleoptera and Hymenoptera. It is important to note this distinction because measures of N or protein content that do not account for exoskeleton can overestimate digestible protein content available to consumers. Additionally, protein content from Lowry and Bradford assays correlated better with metabolizable N than total N, supporting the accuracy of metabolizable measures of nutrient content (Wilder et al., 2019; Wilder & Barnes, In preparation). Interestingly, the two colorimetric protein assays, Bradford and Lowry, also resulted in different patterns of results, likely because each assay interacts with amino acids slightly differently (Ku et al., 2013; Winters & Minchin, 2005). This could mean that N‐based measures, such as metabolizable N, may be less prone to measurement variation caused by differences in amino acid or protein structure between samples.

Lipid content was also highly variable within and between orders of arthropods. It is likely that observed differences in lipid content and variation within and between orders are due in part to the diversity of trophic levels represented by taxa contained therein (Wilder et al., 2013). For example, Coleoptera and Hemiptera are extremely diverse orders that contain detritivores, herbivores, omnivores, and carnivores, and these orders exhibited higher variation in lipid content than any other order. Variation within orders could also result from variation among individuals in their feeding history, sex, developmental stage, and reproductive state (Lease & Wolf, 2011). Unlike exoskeleton and protein, lipids are stored in large quantities and rapidly mobilized for energy (Canavoso et al., 2001), and it is likely that we observed significant variation between individual arthropods based on their feeding history and individual lipid storage reserves.

There was much larger within‐order variation in metabolizable C than total C, particularly in orders with high and variable exoskeleton content (i.e., Coleoptera). Total C content predicted lipid content better than metabolizable C content, though neither measure of C accounted well for variation in lipid content. Elemental C may not be a good predictor of arthropod lipid content because variation in C content stems from three pools in individual arthropods: lipid, exoskeleton, and all other organic compounds, all of which contain C by definition (Lease & Wolf, 2010, 2011). For exoskeleton, total C was a poor predictor of exoskeleton content, but metabolizable C was a strong inverse predictor of exoskeleton. Arthropods that had low exoskeleton content had high metabolizable C content, suggesting that consumers of arthropods gain more metabolizable carbon from prey low in exoskeleton content (Bell, 1990; Weiser et al., 1997).

Prey availability is important to consumers in that it influences nutrient intake. There is evidence for prey selection based on nutrient content in some predators (Araneae; Schmidt et al., 2012) and birds (Kaspari & Joern, 1993). However, bobwhites are thought to be more responsive to fluctuations in relative abundance, as many precocial chicks may not be able to afford selectivity beyond their morphological limitations allow (i.e., small beak gape, low mobility) with such high pressure to grow quickly (Butler et al., 2012; Doxon & Carroll, 2010; Eubanks & Dimmick, 1974; Osborne et al., 2012). Yet, not all prey are equal in their nutrient content. Common arthropod prey vary significantly in the content of nutrients and indigestible components. Consuming prey high in indigestible content likely decreases overall nutrient intake and could have consequences for growth or survival (Hejl & Verner, 1990; Kaspari & Joern, 1993; Miles, 1990; Morrow et al., 2015; Sakai & Noon, 1990). Ongoing declines in grassland arthropods and birds necessitate increased understanding of the interactions that determine the growth and survival of these species (Brennan, 1991; Hernandez et al., 2013; Sanchez‐Bayo & Wyckhuys, 2019). In addition, conditions that alter the community composition of arthropods in ways that shift the relative balance of high versus low exoskeleton prey could have consequences for insectivore growth, even if the overall abundance of prey does not change. For example, Reeves et al. (In revision) found that prescribed burning significantly increased the abundance of Hymenoptera, which have high exoskeleton and low protein content, at the current study site. Our results suggest that measures of food availability for animals that feed on arthropods should consider more than the abundance and diversity of these prey as different orders of arthropods can vary significantly in their nutrient availability and digestibility for consumers.

## CONFLICT OF INTEREST

The authors declare no conflict of interest in this work.

## AUTHOR CONTRIBUTIONS

**Jamie T. Reeves:** Conceptualization (equal); Data curation (equal); Formal analysis (equal); Investigation (equal); Methodology (equal); Writing‐original draft (equal); Writing‐review & editing (equal). **Samuel D. Fuhlendorf:** Conceptualization (equal); Funding acquisition (equal); Methodology (equal); Supervision (equal); Validation (equal); Writing‐review & editing (equal). **Craig A. Davis:** Conceptualization (equal); Funding acquisition (equal); Methodology (equal); Supervision (equal); Validation (equal); Writing‐review & editing (equal). **Shawn M. Wilder:** Conceptualization (equal); Funding acquisition (equal); Investigation (equal); Methodology (equal); Resources (equal); Supervision (equal); Validation (equal); Writing‐original draft (equal); Writing‐review & editing (equal).

### OPEN RESEARCH BADGES

This article has earned an Open Data Badge for making publicly available the digitally‐shareable data necessary to reproduce the reported results. The data is available at [https://doi.org/10.5061/dryad.t76hdr81b].

## Supporting information

Table S1

## Data Availability

The data used in this work are available through Dryad at the following doi: https://doi.org/10.5061/dryad.t76hdr81b.
