# Supplementary material for: Arthropod prey vary among orders in their nutrient and exoskeleton content
Source: Ecol Evol. 2021 Dec 14;11(24):17774–85. doi: 10.1002/ece3.8280 (PMC8717265; doi:10.1002/ece3.8280)
Supplement: Supplementary file 1 — Table S1 [file ECE3-11-17774-s001.docx]

**Supplementary Material**

**Table S1.** Equations and test statistics for linear regressions of total and metabolizable C and N content with Lipid, Exoskeleton, Lowry protein, and Bradford protein content for all orders together and individually. Significant relationships are marked with (*).

| Exoskeleton |  | **a) Total C Content** | | | | **b) Metabolizable C Content** | | | |
| --- | --- | --- | --- | --- | --- | --- | --- | --- | --- |
|  | Coefficient | Equation | DF | R^2^ | p | Equation | DF | R^2^ | p |
|  |  |  |  |  |  |  |  |  |  |
|  | All Orders | y = 1.9x – 64.0 | 70 | 0.1 | 0.004* | y = -2.2x + 102.1 | 70 | 0.9 | < 0.0001* |
|  |  |  |  |  |  |  |  |  |  |
|  | Araneae | y = 0.4x – 11.7 | 3 | 0.05 | 0.4 | y = 0.2x – 2.4 | 3 | 0.06 | 0.7 |
|  | Lepidoptera | y = -1.8x + 89.6 | 3 | 0.07 | 0.7 | y = -2.9 + 97.2 | 3 | 0.9 | 0.02* |
|  | Orthoptera | y = -0.2x + 21.5 | 12 | 0.007 | 0.8 | y = -1.4x + 67.5 | 12 | 0.6 | 0.0006* |
|  | Hemiptera | y= -2.7x + 149.5 | 20 | 0.08 | 0.1 | y = -1.8x + 91.8 | 20 | 0.9 | < 0.0001* |
|  | Hymenoptera | y = -30.6x + 1486.3 | 1 | 0.1 | 0.8 | y = -2.7x + 127.2 | 1 | 0.9 | 0.02* |
|  | Coleoptera | y = -2.1 + 138.0 | 21 | 3.63 | 0.4 | y = -1.9x + 98.0 | 21 | 0.9 | < 0.0001* |
|  |  |  |  |  |  |  |  |  |  |
| Lipid |  | **c) Total C Content** | | | | **d) Metabolizable C Content** | | | |
|  | Coefficient | Equation | DF | R^2^ | p | Equation | DF | R^2^ | p |
|  |  |  |  |  |  |  |  |  |  |
|  | All Orders | y = 1.6x – 59.9 | 70 | 0.3 | < 0.0001* | y = 0.3x – 2.7 | 70 | 0.1 | 0.02* |
|  |  |  |  |  |  |  |  |  |  |
|  | Araneae | y = 0.6x – 17.6 | 3 | 0.2 | 0.5 | y = 0.9x – 27.1 | 3 | 0.01 | 0.4 |
|  | Lepidoptera | y = -1.0x + 49.5 | 3 | 0.3 | 0.2 | y = 0.04x + 7.9 | 3 | 0.006 | 0.9 |
|  | Orthoptera | y = 1.0x – 38.7 | 12 | 0.2 | 0.2 | y = 0.4x – 10.6 | 12 | 0.2 | 0.08 |
|  | Hemiptera | y = 1.0x – 29.5 | 20 | 0.02 | 0.2 | y = 0.5x + 1.9 | 20 | 0.2 | 0.03* |
|  | Hymenoptera | y = -58x + 2775.5 | 1 | 0.8 | 0.2 | y = -0.3x + 28.6 | 1 | 0.002 | 0.9 |
|  | Coleoptera | y = 3.7x – 165.4 | 21 | 0.5 | 0.0002* | y = 0.8x – 9.8 | 21 | 0.4 | 0.001* |
|  |  |  |  |  |  |  |  |  |  |
| Lowry Protein |  | **e) Total N Content** | | | | **f) Metabolizable N Content** | | | |
|  | Coefficient | Equation | DF | R^2^ | p | Equation | DF | R^2^ | p |
|  |  |  |  |  |  |  |  |  |  |
|  | All Orders | y = 3.7x + 1.0 | 70 | 0.2 | 0.0002* | y = 3.7x + 8.4 | 70 | 0.5 | < 0.0001* |
|  |  |  |  |  |  |  |  |  |  |
|  | Araneae | y = 9.6x – 49.3 | 3 | 0.9 | 0.001* | y = 10.9x – 55.3 | 3 | 0.9 | 0.002* |
|  | Lepidoptera | y = 4.3x + 6.4 | 3 | 0.7 | 0.05* | y = 3.0x + 18.2 | 3 | 0.5 | 0.1 |
|  | Orthoptera | y = 1.1x + 33.1 | 12 | 0.06 | 0.4 | y = 1.7x + 28.3 | 12 | 0.08 | 0.2 |
|  | Hemiptera | y = -0.7x + 39.9 | 20 | 0.01 | 0.6 | y = 0.4x + 30.6 | 20 | 0.006 | 0.7 |
|  | Hymenoptera | y = 0.7x + 14.2 | 1 | 0.06 | 0.8 | y = 3.0x + 6.0 | 1 | 0.2 | 0.4 |
|  | Coleoptera | y = 2.6x + 3.1 | 21 | 0.1 | 0.05* | y = -0.5x + 29.4 | 21 | 0.01 | 0.6 |
|  |  |  |  |  |  |  |  |  |  |
| Bradford Protein |  | **g) Total N Content** | | | | **h) Metabolizable N Content** | | | |
|  | Coefficient | Equation | DF | R^2^ | p | Equation | DF | R^2^ | p |
|  |  |  |  |  |  |  |  |  |  |
|  | All Orders | y = 5.2x – 15.3 | 68 | 0.2 | < 0.0001* | y = 3.6x + 7.6 | 68 | 0.3 | < 0.0001* |
|  |  |  |  |  |  |  |  |  |  |
|  | Araneae | y = 3.4x + 24.0 | 3 | 0.6 | 0.09 | y = 4.2 + 18.9 | 3 | 0.7 | 0.05* |
|  | Lepidoptera | y = 11.8x – 66.1 | 1 | 0.2 | 0.4 | y = 3.8x – 9.5 | 1 | 0.06 | 0.5 |
|  | Orthoptera | y = 3.9x – 11.0 | 12 | 0.03 | 0.3 | y = 6.4x – 30.9 | 12 | 0.2 | 0.05* |
|  | Hemiptera | y = 1.7x + 23.7 | 20 | 0.02 | 0.3 | y = 2.0x + 25.1 | 20 | 0.1 | 0.08 |
|  | Hymenoptera | y = 1.1x + 14.8 | 1 | 0.04 | 0.9 | y = 5.4x – 2.2 | 1 | 0.1 | 0.5 |
|  | Coleoptera | y = 3.0x + 0.1 | 21 | 0.04 | 0.2 | y = 4.3x + 3.5 | 21 | 0.3 | 0.006* |
|  |  |  |  |  |  |  |  |  |  |
